# Pertussis clinical case definition: Time for change in developing countries?

**DOI:** 10.1371/journal.pone.0219534

**Published:** 2019-07-10

**Authors:** Sheila Gopal Krishnan, Weng Hong Fun, Malini Devi Ramadras, Rahmah Yunus, Yik Fan Lye, Sondi Sararaks

**Affiliations:** 1 Paediatrics Department, Hospital Kulim, Kedah, Malaysia; 2 Centre For Health Outcomes Research, Institute For Health Systems Research, Selangor, Malaysia; University of Georgia, UNITED STATES

## Abstract

**Background:**

Developing countries still struggle with late detection and mortality from pertussis. A review of clinical case definitions is necessary for early disease detection. This paper aimed to study possible clinical characteristics for earlier pertussis detection in a sporadic setting.

**Methods:**

We conducted a retrospective review of medical and laboratory records in a general paediatric ward of a district hospital in a developing country. Inclusion criteria were all children hospitalised with nasopharyngeal swab taken for *Bordetella pertussis*. We compared sensitivity and specificity of World Health Organization diagnostic criteria with other clinical characteristics. Polymerase chain reaction *Bordetella pertussis* was the gold standard used.

**Results:**

Out of 207 eligible admissions, the study retrieved 128 complete records. Approximately half of the children were less than 3 months old. The World Health Organization diagnostic criteria had a low sensitivity (15%), but high specificity (92%). In comparison, combinations that included paroxysmal cough, ill contact and facial congestion had higher sensitivity. Increasing cough duration improved specificity while compromising sensitivity.

**Conclusion:**

Several clinical characteristics such as paroxysmal cough, facial congestion and a history of ill contact have potential for early clinical detection. Conventional emphasis on cough duration may hamper early detection.

## Introduction

Pertussis remains a major cause of childhood illness with estimated case fatality rates of 4% in developing countries [1]. This disease spares no age group, though infants have the highest casualties [2]. A resurgence in the incidence of pertussis has been observed in the recent years globally [3], and implicated were acellular vaccine effectiveness, asymptomatic transmission from individuals vaccinated with acellular vaccines [4], genetic adaptation of *Bordetella pertussis* [5], vaccination delay or refusal [6], improved surveillance and laboratory capabilities [7] as well as overall increased awareness of the disease [2].

At the ground level, diagnosing pertussis remains a major challenge for clinicians. Variability in definitions, evolving clinical spectrum from prior vaccination and lack of classical symptoms [8] complicate the presentation. Most clinical case definitions require a two-week cough with at least one additional symptom of paroxysmal cough, inspiratory whoop, posttussive vomiting or apnoea [9]. However, exceptions exist. France requires cough of more than 7 days with at least one symptom of paroxysmal cough, whooping, or vomiting, and a high index of suspicion if clinical presentation is at early infancy [9]. Case definitions that rely heavily on a duration of 2 weeks are useful in an outbreak situation, however its reliability for early diagnosis of sporadic pertussis cases is not well documented [8].

Although the incidence of pertussis, a notifiable illness in Malaysia, was less than 1/100,000 population prior to 2012 with no deaths in the preceding decade [10, 11], a mortality rate of 0.01/100,000 (two deaths) was observed in 2012 [12, 13]. National vaccination coverage for diphtheria, pertussis and tetanus (DPT), based on live births data, was 93.6% and 97.3% for first and third dose respectively; coverage was similar for 2012 [12, 13]. In the state where the study was conducted, vaccination coverage was 90.9% and 92.7% for first and third dose respectively in 2011, with similar coverage in 2012 [12, 13]. With changes in epidemiology of pertussis in the past few decades, use of established definitions may not effectively detect disease in the early stages [14, 15]. Therefore, we aim to study possible clinical characteristics for earlier detection.

## Methods

### Design

In 2013–2014, using the hospital laboratory list, we traced hospital registration numbers (RN) of all who had nasopharyngeal swab samples sent for polymerase chain reaction (PCR) for *Bordetella pertussis* (PCRBp) from January 2011 to December 2012. We used the RN to trace medical records and pharmacists and senior house officers performed data extraction. The authors matched the PCRBp results, retrieved from hospital laboratory records, with data extracted from medical records, using RN.

We excluded children who upon admission required intensive care, or those with confirmed immunodeficiency syndrome. We trained data collectors on the use of a structured pretested data collection form to improve data reliability. We collected individual characteristics, risk factors, clinical presentation, signs and symptoms, antibiotic management and outcome.

### Setting

The chosen site was a 26-bedded general paediatric ward with two resident paediatricians in a district hospital in northern region of Peninsular Malaysia that recorded high numbers of pertussis cases. The hospital practice was to send samples for PCRBp for all presumed pertussis cases as well as part of a differential diagnosis workup. In 2011–2012, PCRBp was ordered for children presenting with a differential diagnosis of pertussis. Hence, the children with PCRBp ordered could likely have other respiratory illness such as pneumonia due to other aetiology. The differential diagnosis of pertussis, although guided by the WHO diagnostic criteria, varied among clinicians.

Of total paediatric admissions in 2011 and 2012, 33.4% had respiratory illness. Of these, 4.3% and 7.8% had a presumptive diagnosis of pertussis in the ward for 2011 and 2012 respectively. Of total respiratory admissions, presumptive pertussis was 5.9% while PCRBp was ordered for 9.6% (S1 Fig).

### Outcomes

Malaysia follows the World Health Organization (WHO) definition for case detection [1, 10]. The WHO diagnostic criteria (WDC) specifies pertussis to be a case diagnosed by a physician or the combination of cough for at least 2 weeks with at least one of the following: prolonged/paroxysmal coughing, inspiratory whooping or posttussive vomiting, without other apparent cause, together with laboratory criteria of *Bordetella pertussis* isolation, a PCR assay or positive paired sera [1]. Malaysia uses the clinical features for diagnosis. We compared the WDC with several other clinical characteristics used in local setting. We specified one combination a priori (cough of at least 3 days duration with paroxysmal cough, with at least one of the following; apnoea, facial congestion, cyanosis, posttussive vomiting or sleep disturbances), and empirically explored other combinations.

The gold standard used was PCRBp, detection of genomic sequences by means of the polymerase chain reaction for *Bordetella pertussis*, using nasopharyngeal swab samples in Amies clear transport medium (clear or without charcoal) sent to a central national public health laboratory (S1 Text) [16]. We compared sensitivity and specificity of clinical characteristic combinations. We excluded symptoms/signs seen in less than 5% of records.

### Sample size, data management and statistics

We captured the RN in the data collection form for merging with laboratory data, and removed this identifier after data cleaning and verification. Of the 15 records without documented outcomes at discharge, 12 were referred for higher-level care. We checked national pertussis mortality statistics to identify possible mortalities as Malaysia requires mandatory reporting on suspected or confirmed pertussis within 7 days [17]. No deaths were reported in the region of the study hospital (northern region, Peninsular Malaysia), with two deaths nationwide, in East Malaysia (Sabah) [13].

Based on expected sensitivity and specificity of 50%, a prevalence of 5%, the minimum sample required was 96; a review of medical records over a two-year period could achieve this [18]. Sensitivity and specificity with a 95% confidence interval (CI) were calculated using standard formulas [19] and subsequently compared. Delay in vaccination, a known risk factor [20], was not computed due to high missing values. Logistic regression was used for odds ratio calculations and modelling for prediction of disease using SPSS version 23.

### Ethics

This study obtained ethical approval for medical and laboratory records review, with waiver for informed consent of parents or legal guardians of the patients, from the Medical Research Ethics Committee Malaysia (letter dated 7 June 2013; Ref(2) dlm. KKM/NIHSEC/800 2/2/2 Jld.2.P13-473). The study was registered in the National Medical Research Register (www.nmrr.gov.my), NMRR-12-1338-14149, and funded by the Ministry of Health Malaysia.

## Results

**Fig 1:**
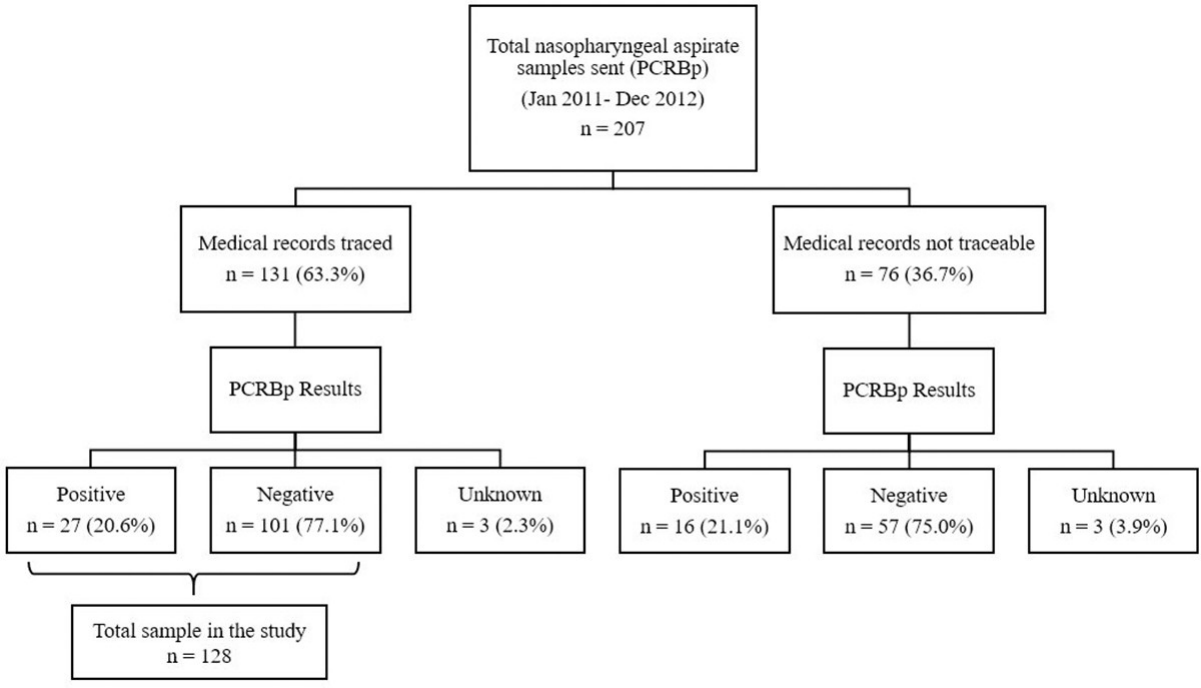
Comparison of medical records traced and PCRBp results.

Of 207 laboratory nasopharyngeal swab samples sent for PCRBp, we retrieved 131 medical records. Of these, 128 had PCRBp results. Of the 128 with PCRBp, 27 (20.6%) were positive. Similarly, among the medical records that could not be traced, 16 (21.1%) were positive for PCRBp (Fig 1).

In the sample, 68% were less than 6 months old, with a male: female ratio of 1.5. Vaccination status was not documented for 59.4%, of which 56.6% was less than 3 months old. (Table 1). The WDC had a lower sensitivity (15%) than most of the other combined clinical characteristics assessed. However, specificity of the WDC was 92% (Table 2).

**Table 1:** Clinical characteristics and determinants of pertussis infection.

Clinical Characteristic	PCR Result		
	Positive	Negative	Total
	number (%)	number (%)	number (%)
**Gender**			
Male	16 (59.3)	61 (60.4)	77 (60.2)
Female	11 (40.7)	40 (39.6)	51 (39.8)
**Age group**			
< 2 months	6 (22.2)	22 (21.8)	28 (21.9)
2 - < 3 months	10 (37.0)	22 (21.8)	32 (25.0)
3 - < 6 months	8 (29.6)	19 (18.8%)	27 (21.1)
≥ 6 months	3 (11.1)	38 (37.6%)	41 (32.0)
**Vaccine Status**			
1st Dose	6 (22.2)	15 (14.9)	21 (16.4)
2nd Dose	2 (7.4)	9 (8.9)	11 (8.6)
3rd Dose	3 (11.1)	17 (16.8)	20 (15.6)
Unknown^a^	16 (59.3)	60 (59.4)	76 (59.4)
**Contact with illness**			
No	5 (18.5)	43 (42.6)	48 (37.5)
Household	21 (77.8)	45 (44.6)	66 (51.6)
Others	1 (3.7)	12 (11.9)	13 (10.2)
Unknown	0 (0)	1 (1.0)	1 (0.8)
**Fever**			
Yes	16 (59.3)	71 (70.3)	87 (68.0)
No	11 (40.7)	29 (28.7)	40 (31.2)
Unknown	0 (0)	1 (1.0)	1 (0.8)
**Poor oral Intake**			
No	18 (66.7)	61 (60.4)	79 (61.7)
Yes	8 (29.6)	38 (37.6)	46 (35.9)
Unknown	1 (3.7)	2 (2.0)	3 (2.3)
**Previous antibiotic treatment**			
No	21 (77.8)	57 (56.4)	78 (60.9)
Yes	6 (22.2)	43 (42.6)	49 (38.3)
Unknown	0 (0)	1 (1.0)	1 (0.8)
**Family members treated**			
No	12 (44.4)	63 (62.4)	75 (58.6)
Yes	14 (51.9)	35 (34.7)	49 (38.3)
Unknown	1 (3.7)	3 (3.0)	4 (3.1)
**Mortality**			
Yes	0 (0)	0 (0)	0 (0)
No	25 (92.6)	88 (87.1)	113 (88.3)
Unknown^b^	2 (7.4)	13 (12.9)	15 (11.7)

^a^Of the 76 with undocumented vaccine status, 43 (56.6%) children were below 3 months old.

^b^Of the 15 labelled “unknown”, no outcome was documented for 3 patients at discharge while 12 were referred for higher level care. National pertussis mortality surveillance statistics (2011–2012) showed no deaths in the region–likely there were no deaths in this sample.

**Table 2:** Sensitivity, specificity and predictive values of pertussis.

	Sensitivity (95% CI)	Specificity (95% CI)	Positive Predictive Value (95% CI)	Negative Predictive Value (95% CI)
**WHO Criteria:**	0.15 (0.05, 0.35)	0.92 (0.84, 0.96)	0.33 (0.11, 0.65)	0.80 (0.71, 0.86)
Cough for >14 days with at least one of the following:				
a) Paroxysmal coughing				
b) Posttussive vomiting without other apparent cause				
**Clinical case Definitions**				
**Cough**				
Cough for >14 days	0.15 (0.05, 0.35)	0.92 (0.84, 0.96)	0.33 (0.11, 0.65)	0.80 (0.71, 0.86)
Cough for >7 days	0.26 (0.11, 0.46)	0.87 (0.78, 0.93)	0.35 (0.15, 0.59)	0.81 (0.72, 0.88)
Cough for ≤7 days	0.74 (0.54, 0.89)	0.13 (0.07, 0.22)	0.19 (0.12, 0.28)	0.65 (0.41, 0.85)
Cough ≥3 days	0.78 (0.58, 0.91)	0.33 (0.24, 0.43)	0.24 (0.16, 0.35)	0.84 (0.69, 0.94)
**Nature of Cough**				
Paroxysmal Cough	0.74 (0.53, 0.88)	0.44 (0.34, 0.54)	0.26 (0.17,0.38)	0.86 (0.73, 0.94)
Cough for >14 days and paroxysmal cough	0.11 (0.02, 0.29)	0.93 (0.86, 0.97)	0.30 (0.07, 0.65)	0.79 (0.70, 0.86)
Cough for >7 days and paroxysmal cough	0.19 (0.07, 0.39)	0.89 (0.80, 0.94)	0.31 (0.12, 0.59)	0.80 (0.71, 0.87)
Cough for ≤7 days and paroxysmal cough	0.56 (0.35, 0.75)	0.55 (0.44, 0.65)	0.25 (0.15, 0.38)	0.82 (0.70, 0.90)
Cough for ≥3 days and paroxysmal cough	0.59 (0.39, 0.77)	0.54 (0.43, 0.64)	0.26 (0.16, 0.39)	0.83 (0.70, 0.91)
**Clinical signs**				
Cough >14 days, paroxysmal cough and ill contact^a^	0.11 (0.03, 030)	0.97 (0.91, 0.99)	0.50 (0.14, 0.86)	0.80 (0.71, 0.86)
Cough >7 days, paroxysmal cough and ill contact	0.19 (0.07, 0.39)	0.96 (0.89, 0.99)	0.56 (0.23, 0.85)	0.81 (0.72, 0.87)
Cough ≤7 days, paroxysmal cough and ill contact	0.44 (0.25, 0.65)	0.72 (0.62, 0.81)	0.31 (0.17, 0.48)	0.82 (0.73, 0.90)
Cough ≥3 days, paroxysmal cough and Ill contact	0.52 (0.32, 0.71)	0.74 (0.64, 0.82)	0.36 (0.22, 0.53)	0.85 (0.75, 0.91)
Cough >14 days, paroxysmal cough and facial congestion	0.07 (0.01, 0.26)	0.95 (0.88, 0.98)	0.29 (0.05, 0.70)	0.79 (0.70, 0.85)
Cough >7 days, paroxysmal cough and facial congestion	0.15 (0.05, 0.35)	0.92 (0.84, 0.96)	0.33 (0.11, 0.65)	0.79 (0.71, 0.86)
Cough ≤7 days, paroxysmal cough and facial congestion	0.52 (0.32, 0.71)	0.63 (0.52, 0.72)	0.28 (0.17, 0.43)	0.82 (0.71, 0.90)
Cough ≥3 days, paroxysmal cough and facial congestion	0.52 (0.32, 0.71)	0.63 (0.52, 0.72)	0.28 (0.16, 0.42)	0.82 (0.72, 0.90)
Cough >14 days, paroxysmal cough, ill contact and facial congestion	0.07 (0.01, 0.24)	0.98 (0.93, 1.00)	0.50 (0.07, 0.93)	0.79 (0.71, 0.86)
Cough >7 days, paroxysmal cough, ill contact and facial congestion	0.15 (0.04, 0.34)	0.97 (0.91, 0.99)	0.57 (0.18, 0.90)	0.80 (0.72, 0.87)
Cough ≤7 days, paroxysmal cough, ill contact and facial congestion	0.41 (0.22, 0.61)	0.77 (0.68, 0.85)	0.33 (0.18, 0.52)	0.82 (0.73, 0.90)
Cough ≥3 days, paroxysmal cough and ill contact and facial congestion	0.44 (0.25, 0.65)	0.78 (0.69, 0.86)	0.36 (0.20, 0.55)	0.84 (0.74, 0.90)
Paroxysmal Cough and Ill contact	0.63 (0.42, 0.80)	0.68 (0.58, 0.77)	0.35 (0.23, 0.51)	0.87 (0.77, 0.93)
Paroxysmal Cough and apnoea	0.00 (0.00, 0.16)	0.98 (0.92, 1.00)	0.00 (0.00, 0.80)	0.77 (0.69, 0.84)
Paroxysmal cough and facial congestion	0.67 (0.46, 0.83)	0.55 (0.45, 0.65)	0.29 (0.18, 0.42)	0.86 (0.74, 0.93)
Paroxysmal cough and cyanosis	0.11 (0.03, 0.30)	0.97 (0.91, 0.99)	0.50 (0.14, 0.86)	0.80 (0.72, 0.87)
Paroxysmal cough and posttussive vomiting	0.37 (0.20, 0.58)	0.74 (0.64, 0.82)	0.28 (0.15, 0.45)	0.81 (0.71, 0.88)
Paroxysmal cough and sleep disturbance	0.15 (0.05, 0.35)	0.83 (0.74, 0.90)	0.19 (0.06, 0.43)	0.78 (0.69, 0.85)
Paroxysmal cough, Ill contact and facial congestion	0.56 (0.36, 0.74)	0.75 (0.65, 0.83)	0.38 (0.23, 0.54)	0.86 (0.77, 0.92)
Cough of at least 3 days duration with paroxysmal cough, with at least one of the following: apnoea, facial congestion, cyanosis, posttussive vomiting or sleep disturbances (a priori combination)	0.59 (0.39, 0.77)	0.57 (0.46, 0.67)	0.28 (0.17, 0.41)	0.83 (0.72, 0.91)
Cough of at least 3 days duration with paroxysmal cough, with at least one of the following: apnoea, facial congestion, cyanosis, posttussive vomiting, sleep disturbances or ill contact	0.52 (0.32, 0.71)	0.74 (0.63, 0.82)	0.36 (0.22, 0.53)	0.84 (0.74, 0.91)
Paroxysmal cough with at least one of the following: apnoea, facial congestion, cyanosis, posttussive vomiting or sleep disturbances	0.74 (0.54, 0.89)	0.48 (0.38, 0.58)	0.28 (0.18, 0.40)	0.87 (0.75, 0.95)
Paroxysmal cough and absence of fever with at least one of the following: apnoea, facial congestion, cyanosis, posttussive vomiting or sleep disturbances	0.37 (0.19, 0.58)	0.84 (0.75, 0.90)	0.38 (0.24, 0.55)	0.83 (0.78, 0.87)
Paroxysmal cough with at least one of the following: apnoea, facial congestion, cyanosis, posttussive vomiting, sleep disturbances or Ill contact	0.74 (0.54, 0.89)	0.45 (0.35, 0.56)	0.27 (0.18, 0.39)	0.86 (0.74, 0.94)
Paroxysmal cough and absence of fever with at least one of the following: apnoea, facial congestion, cyanosis, posttussive vomiting, sleep disturbances or Ill contact	0.37 (0.19, 0.58)	0.84 (0.75, 0.90)	0.38 (0.24, 0.55)	0.83 (0.78, 0.87)

^a^Ill contact = history of household member with respiratory illness

Analysis of cough duration showed cough of >14 days had a higher specificity with low sensitivity compared to other cough durations analysed. Cough duration of ≥3 days and ≤7 days did not substantially increase sensitivity and specificity (Table 2).

The presence of paroxysmal cough alone had a sensitivity of 74% with specificity of 44%. The addition of ill contact and facial congestion resulted in lower sensitivity (56%) and higher specificity (75%). When the above three symptoms were combined with cough of >14 days, specificity is high at 98% with a sensitivity of 7%. The addition of apnoea, cyanosis, posttussive vomiting or sleep disturbance to paroxysmal cough improved specificity, however only facial congestion showed higher sensitivity and increased likelihood of illness. The combination of absence of fever and paroxysmal cough with at least one other symptom improved specificity at the cost of sensitivity (Table 2). Additionally, age and history of ill contact significantly predicted confirmed pertussis, while symptoms found to contribute to the model were facial congestion, cyanosis and sleep disturbances. (Table 3).

**Table 3:** Univariate and multivariable analysis.

Clinical Characteristic	PCR Result (n = 128)		Univariate Analysis	Multivariable Analysis^a^
	Positive	Negative		
	number (%)	number (%)	OR (95% CI)	OR (95% CI)
**Cough Duration**				
< 3 days	6 (22.2)	32 (31.7)	1	
3–7 days	14 (51.9)	53 (52.5)	1.41 (0.49–4.04)	-
8–14 days	3 (11.1)	5 (5.0)	3.20 (0.60–17.10)	-
> 14 days	4 (14.8)	8 (7.9)	2.67 (0.61–11.76)	-
Unknown^b^	0 (0.0)	3 (3.0)	-	-
**Paroxysmal cough**				
No	7 (25.9)	44 (43.6)	1	-
Yes	20 (74.1)	56 (55.4)	2.25 (0.87–5.79)	-
Unknown^b^	0 (0)	1 (1.0)	-	
**Facial congestion**				
No	6 (22.2)	44 (43.6)	1	1
Yes	21 (77.8)	57 (56.4)	2.70 (1.01–7.26)	2.64 (0.92–7.63)
**Apnoea**				
Yes	1 (3.7)	5 (5.0)	1	-
No	26 (96.3)	90 (89.1)	1.44 (0.16–12.92)	-
Unknown^b^	0 (0)	6 (5.9)	-	
**Cyanosis**				
No	23 (85.2)	95 (94.1)	1	1
Yes	4 (14.8)	6 (5.9)	2.75 (0.72–10.57)	3.59 (0.69–18.56)
**Posttussive vomiting**				
No	15 (55.6)	60 (59.4)	1	-
Yes	12 (44.4)	41 (40.6)	1.17 (0.50–2.76)	-
**Sleep disturbance**				
Yes	5 (18.5)	26 (25.7)	1	1
No	22 (81.5)	75 (74.3)	1.53 (0.52–4.44)	2.12 (0.61–7.41)
**Age**				
<2 months	6 (22.2)	22 (21.8)	3.46 (0.79–15.21)	1.33 (0.25–7.19)
2 - <3months	10 (37.0)	22 (21.8)	5.76 (1.43–23.18)	3.61 (0.82–15.96)
3 - <6 months	8 (29.6)	19 (18.8)	5.33 (1.27–22.44)	4.14 (0.89–19.20)
≥ 6 months	3 (11.1)	38 (37.6)	1	1
**History of Ill Contact**				
No	5 (18.5)	43 (42.6)	1	1
Household	21 (77.8)	45 (44.6)	4.01 (1.39–11.60)	3.19 (1.00–10.20)
Others	1 (3.7)	12 (11.9)	0.72 (0.08–6.73)	0.60 (0.06–6.32)
Unknown^b^	0 (0.0)	1 (1.0)	-	-

^a^Omitted variables that did not significantly contribute to the final model.

^b^Excluded from analysis

^c^Final model consisted of facial congestion, cyanosis, sleep disturbance, age and history of ill contact.

## Discussion

Certain combinations of clinical characteristics had higher sensitivity. In contrast, the WDC achieved high specificity. Although paroxysmal cough, cyanosis, apnoea, posttusssive vomiting, sleep disturbance, history of ill contact and absence of fever were specific, facial congestion was an important clinical symptom that increased sensitivity as well. Paroxysmal cough, with or without ill contact and facial congestion, seems to be promising for early detection. The use of these signs/symptoms may be more effective, even when cough duration was omitted.

A study in South Africa showed that by adding apnoea to the WHO criteria, sensitivity increased from 31% to 55%, while sensitivity increased further to 84% when duration of cough was omitted [21]. Results from this study supports omission of cough duration; the nature of cough, i.e. paroxysmal, rather than cough duration, achieved a higher sensitivity, as noted previously [22]. Furthermore, the use of cough duration (2 weeks) as the main criteria may delay diagnosis and treatment of children who presented early.

Besides increasing specificity, ill contact history was a predictor of illness in this study. Similar to results seen here, two thirds of subjects in South Africa had an ill contact, either a parent or sibling [21]; in the United States, an ill contact was identified in 43% of subjects, the majority of whom were adults [2]. Crowcroft NS *et al*. [23] found that casual contact from the community was responsible for 34% of pertussis transmission to young infants while Zouari A *et al*. [24] noted mothers as a likely source of infection.

The occurrence of cyanosis, sleep disturbance and apnoea were not common in this study, whereas facial congestion appeared to be an important clinical sign; Vesselinova-Jenkins *et*. *al*. had noted a higher prevalence of facial congestion compared to the other symptoms [25]. Furthermore, pertussis was more likely to occur in age less than 6 months, similar to Northern Iran and Spain [26, 27]. Studies elsewhere showed that clinical presentations varied across regions [26, 28]. Compared to individual symptoms, modelling with multiple symptoms for disease prediction has not been successful for children [29], as seen in this study.

This study involved data from one centre and selected cases with PCRBp. The identification of these cases did not rely on a disease reporting mechanism. This study did not look at the proportion of children with a differential of pertussis who did not get PCRBp test. Ideally, all children with respiratory tract infection should be the study population; however, cost was a limiting factor. The sample in this study were children with mild to moderate illness, as excluded were those who were admitted into intensive care. We did not use culture, the gold standard for pertussis diagnosis [30], as it was not a common practice at the study site.

We were unable to trace a third of the medical records despite repeated efforts. Additionally, documentation completeness, such as information on PCR results and vaccination status could affect study results. Furthermore, healthcare providers used prolonged and paroxysmal cough interchangeably; we accepted either term to mean paroxysmal cough in this study. Clinicians routinely asked for a history of household ill contact for all children presenting with respiratory illness. However, recall bias is possible.

Facial congestion in the study refers to facial discolouration due to venous congestion whereby child goes red or blue in the face [31, 32]; and unless perceived to be of clinical relevance, might not be documented. However, medical records without facial congestion documented did not necessarily mean that it was absent. The documentation of facial congestion implied either mere documentation of a sign detected, or a perceived medical significance. Facial congestion is likely a milder sign compared to cyanosis, possibly explaining the lower occurrence of cyanosis in this study. The two may reflect different spectrum of severity.

It is not uncommon for general practitioners in the study region to prescribe macrolides (erythromycin/azithromycin) [33–35]; this may affect illness trajectory and PCRBp positivity. This study did not explore the type or effect of previous antibiotics treatment, nor challenges related to surveillance or reporting of pertussis cases. Fever, typically low grade or absent [36, 37] was high in this study. However, it was not a practice to do a viral multiplex PCR test concomitantly with PCRBp for co-infection in the study hospital as was reported elsewhere [38]. The issues above, as well as factors influencing the decision for PCRBp, including variability in diagnostic decision-making are areas for future exploration.

Internationally, although prevention initiatives such as maternal vaccination was explored, costs may be a hurdle locally. Findings from this study suggest that several clinical characteristics have the potential to increase the chance of earlier diagnosis. To facilitate early detection and control, an effective screening tool needs a higher sensitivity, sacrificing specificity [9]. Key clinical features for a suspected pertussis case were paroxysmal cough, a history of ill contact and facial congestion. Cough duration increased specificity while lowering sensitivity; that is likely to prevent early detection. Clinicians could consider utilising these findings for earlier pertussis detection. Further studies could improve the validity of suggested clinical characteristics and its applicability; this could improve pertussis screening without burdening the healthcare system. Therefore, the challenge of recognising this disease remains a continual one.

## Conclusions

Changes in clinical case definitions could improve the early detection of pertussis, and contribute to reducing spread of illness, complications and mortality. Although, prevention is undisputedly superior, key clinical features that may facilitate early detection include nature of cough, facial congestion and history of ill contact, while cyanosis, apnoea and sleep disturbance increased diagnostic accuracy. Shifting the emphasis away from cough duration as the hallmark of pertussis infection may be necessary.

## Supporting information

S1 Dataset Pertussis dataset. (XLSX)

S1 Fig Ward 2 admission statistics, 2011–2012. (PDF)

S1 Text Summary of laboratory method for PCRBp. (DOCX)
