# Supplementary material for: Pertussis clinical case definition: Time for change in developing countries?
Source: PLoS One. 2019 Jul 10;14(7):e0219534. doi: 10.1371/journal.pone.0219534 (PMC6619773; doi:10.1371/journal.pone.0219534)
Supplement: S1 Fig — (PDF) [file pone.0219534.s002.pdf]

S1 Fig. Ward 2 Admission Statistics, 2011-2012

STATISTIK KEMASUKAN PESAKIT KE WAD 2 BAGI TAHUN 2011
WARD 2 ADMISSION STATISTICS FOR YEAR 2011

BULAN *Month*	JAN	FEB	MAR	APR	MAY	JUN	JUL	AUG	SEPT	OCT	NOV	DEC
JUMLAH *Total*	287	262	273	299	295	296	286	240	312	328	316	273

JUMLAH KES RESPIRATORI: 1176
Total Respiratory Case: 1176

JUMLAH KES PERTUSIS: 51 (4.3%*)
Total Pertussis Case: 51 (4.3%)*

STATISTIK KEMASUKAN PESAKIT KE WAD 2 BAGI TAHUN 2012
WARD 2 ADMISSION STATISTICS FOR YEAR 2012

BULAN *Month*	JAN	FEB	MAR	APR	MAY	JUN	JUL	AUG	SEPT	OCT	NOV	DEC
JUMLAH *Total*	304	311	324	271	252	194	201	169	206	268	264	210

JUMLAH KES RESPIRATORI: 975
Total Respiratory Case: 975

JUMLAH KES PERTUSIS: 76 (7.8%*)
Total Pertussis Case: 76 (7.8%)*

*Total Pertussis/Total Respiratory Case
