# Supplementary material for: Pertussis clinical case definition: Time for change in developing countries?
Source: PLoS One. 2019 Jul 10;14(7):e0219534. doi: 10.1371/journal.pone.0219534 (PMC6619773; doi:10.1371/journal.pone.0219534)
Supplement: S1 Text — (DOCX) [file pone.0219534.s003.docx]

**S1 Text. Summary of Laboratory Method for PCRBp.**

**DNA extraction**

DNA was extracted from 200ul samples using Qiagen QIAmp DNA Mini Kit (Qiagen, Hamburg GmbH, Germany) and prepared according to manufacturer’s instructions. Genomic DNA extractions were stored under -20°C or -80°C for long term storage.

**PCR Amplification**

The presence of *Bordetella pertussis* was determined using two real-time PCR assay [1]: detection of insertion sequence (IS) 481 region and Pertussis toxin promoter region (ptxA).

A 145-bp of IS481 region was amplified using (BP-1) 5’ – GAT TCA ATA GGT TGT ATG CAT GGT T; (BP-2) 5’ – TTC AGG CAC ACA AAC TTG ATG GGC G; Probe 1 (BP-1) 5’ – TCG CCA ACC CCC CAG TTC ACT CA-(F) and Probe 2 (BP-2) 5’ – AGC CCG GCC GGA TGA ACA CCC-(P) using LightCycler® 480 (Roche). DNA of *B. pertussis* ATCC 8467 was used as positive control.

WHO modified method [2] for Pertussis detection was used. 20ul of master mixed solution (Nuclease Free Water 8ul); PCR premix (LightCycler®Fast Start DNA MasterPLUS HybProbe) 4ul; 1ul each of 10umol BP-1 and BP-2, 0.5ul of 2uM BP-1 and BP-2 probe were mixed with 15ul of DNA sample.

The procedure consisted of 10 cycles of denaturation at 94°C for 10s, annealing at 60°C for 20s and extension at 72°C for 20s for 40 cycles.

All Bordetella positive screenings were confirmed by the same protocol using PT-1 CCA ACG CGC ATG CGT GCA GAT TCG TC and PT-2 CCC TCT GCG TTT TGA TGG TGC CTA TTT TA. A positive result will show an amplified product of 181–bp of pertussis toxin region. DNA of *B. pertussis* ATCC 8467 was used as positive control.

**Results Interpretation**

The target DNA sequence for the laboratory diagnosis of whooping cough using PCR is located in the insertion sequence 481 (IS*481*) and PT promoter (pertussis toxin promoter region, *ptxA-*Pr). A positive IS*481* PCR can be considered as a probable *B. pertussis* infection, when the clinical symptoms are in accordance with this result. In the case of unknown clinical data, positive results from an IS*481* PCR should only be regarded as evidence of infection with *Bordetella* sp. The use of *ptxA-*Pr*,* the single copy number target *B. pertussis*-specific assay will further confirm *B. pertussis* DNA detection.

*Note:*

Summary prepared by Dr. Wan Noraini Wan Yussof, Head of Bacteriology Section, National Public Health Laboratory, Ministry of Health Malaysia.

**References**

1. Dalby T, Krogfelt K, Wirsing von Koenig C, Riffelmann M, Guiso N, Guillot S, et al. Guidance and protocol for the use of real-time PCR in laboratory diagnosis of human infection with Bordetella pertussis or Bordetella parapertussis. ECDC Technical Report, 2012.

2. World Health Organization. Laboratory Manual for the diagnosis of Whooping Cough caused by *Bordetella pertussis/ Bordetella parapertussis* Geneva: 2007.
